# On the socio-cognitive coercion mechanism of the formation of mandarin construction “V+ta(he)+XP”

**DOI:** 10.3389/fpsyg.2025.1635740

**Published:** 2025-09-26

**Authors:** Pan Zhang

**Affiliations:** School of Foreign Languages, Chengdu University, Chengdu, China

**Keywords:** the “V+ta+XP” construction, the “V+ta x de+XP” structure, socio-cognitive coercion, “De vulgarization”, cognitive sociolinguistics

## Abstract

This study employs a cognitive-sociolinguistic framework to investigate the formation mechanism of the Mandarin single-object construction “V+Ta+XP” and the grammaticalization of the non-referential pronoun “Ta.” Building on the hypothesis that “Ta” originates from the abbreviation of the expletive phrase “Ta x de,” we conduct a comparative analysis of the syntactic, pragmatic, and socio-cognitive features of the “V+Ta+XP” construction and its precursor “V+Ta x de+XP” structure. Drawing on 402 “V+Ta x de+XP” and 607 “V+Ta+XP” instances extracted from the CCL and BCC corpora, our findings reveal systematic parallels in syntactic configurations and pragmatic functions, alongside critical socio-cognitive divergences. Specifically, the “V+Ta+XP” construction emerges as a de-vulgarized variant of “V+Ta x de+XP,” driven by socio-cognitive coercion—a process that suppresses vulgar components “x de” to mitigate face-threatening effects while preserving syntactic integrity and emotional salience. By integrating construction grammar, politeness theory, and sociolinguistic variation, this study demonstrates how social norms (e.g., register formality, gender dynamics) and cognitive-emotional needs jointly shape grammaticalization. The proposed framework advances coercion theory by highlighting the interplay of linguistic structure, pragmatic adaptation, and socio-cultural constraints, offering novel insights into the evolution of non-referential elements in Mandarin and their role in balancing expressive force with social acceptability.

## 1 Introduction

The Mandarin “V+Ta+XP” construction, a focus of linguistic debate, exhibits syntactic and semantic features distinct from typical double-object constructions. Examples like (1)–(3) illustrate this structure:

(1) 打他一宿麻将

dǎ tā yīxiǔ májiàng

Da [Ta] yixiu majiang

play TA mahjong all night.

(2) 逛他三天北京城

Guàng tā sāntiān běijīngchéng

Guang [Ta] santian beijingcheng

Visite TA Beijing for three days.

(3) 喝他个痛快

Hē tāgè tòngkuài

He [Ta] ge tongkuai

Drink TA delightedly.

While syntactically resembling double-object constructions, “Ta” here is non-referential and does not contribute to propositional meaning, aligning with “emotive” or “expressive” functions (Jakobson, 1960; Potts, 2005, 2007). Previous studies classify this structure as either a single-object or atypical double-object construction, with “Ta” labeled as a “dummy indirect object” (Zhao, 1979), “virtual reference object” (Zhu, 1982; Ma, 1983), or “formal object, dummy object” (Lv, 1985; Yuan, 2003). Lv (1985, p. 29) pointed out that in syntactic distribution, “Ta” appears after the verb and occupies the object’s position. It can either follow the intransitive verb and act as a dummy object, forming a dative-object structure, e.g., *Pinbo Ta jinian* “fighting TA some years”; or it can be inserted between the real dative-object structure, creating a double-object structure, e.g., *mai Ta liangjin yangrou* “buying TA two jin of mutton”; but it cannot be inserted between the real double-object structure. Yuan (2003) argued that “since the syntactic status of this ‘Ta’ is an object, and there is no triple-object structure in Chinese; so it is natural that a further formal object ‘Ta’ is not allowed to be inserted in the double-object structure.” As to why “Ta” is inserted in the mono-object structure, from the point of view of rhyming syntax, Yuan (2003) argued that “Ta” herein is non-referential, and the insertion of “Ta” can form a rhyming word with the preceding monosyllabic verb, forming a rhyming feature. However, there are “V+*Ta*+XP” constructions guided by two-syllable verbs (phrases), such as *Zefa Ta ge Tongkuai* “punish Ta delightedly.” If the existence of “Ta” is only the result of the rhyme mechanism, then why do verbs already bi-syllabic still need a “Ta” to make up the syllables?

From the perspective of cognitive linguistics, Wang (2008) believes that slightly different from the general ditransitive construction, the agent in the ditransitive construction “V+*Ta*+XP” expects to “transfer” not an entity, but an action, an event, which is a relatively abstract concept. For example, in *Guang Ta liangtian Beijingcheng* “visiting Beijing for two days,” the agent hopes to obtain the two aspects of “visiting Beijing for two days” and “visiting Beijing” in the whole event of “visiting Beijing for two days.” Wang and Wang (2009) believes that the construction inherits relevant semantic, syntactic, and pragmatic features from “single object construction,” “double object construction,” “tone construction,” “mood construction,” “verb complement construction” and other constructions. However, what is the formation mechanism of “Ta” when “Ta” is non-referential? Why does the structure express emotional catharsis? In addition, Wang has mentioned “mood construction,” but what does “mood construction” mean? This is a difficult question to answer because it cannot be uniformly regulated in syntax.

Contrary to this line of argument, scholars such as Huang and Liao (2003), He (2010), Zhang (2011), Zhong (2015), Yu and Zhao (2023), and Zheng (2023) argue that the virtual reference pronoun *Ta* in this construction is no more than a pragmatic component expressing the emotional meaning of the agent, rather than a syntactic component. Put another way, they claim that the structure should be syntactically a single-object one. Scholars like Xiong (2006) and Wang (2009) hypothesize that “Ta” originates from the abbreviation of the expletive phrase “Ta x de” (e.g., Ta ma de, Ta niang de, Ta mei de, etc.), conveying some special emotions. This study tests this hypothesis by comparing the “V+Ta+XP” construction with its precursor “V+Ta x de+XP” structure across syntactic, semantic, pragmatic, and socio-cognitive dimensions. Through corpus analysis of 402 “V+Ta x de+XP” structures and 607 “V+Ta+XP” constructions from the CCL and BCC corpora, we address:

1. How do the “V+Ta+XP” construction and “V+Ta x de+XP” structure align in syntactic and pragmatic features?
2. What socio-cognitive factors drive the deletion of “x de”?
3. What mechanisms underlie the formation of the “V+Ta+XP” construction?

Previous studies on the “V+*Ta*+XP” construction have been mostly static; it is rarely analyzed from the perspective of dynamic communicative processes or verbal expression strategies. The present study therefore focuses on the influence of the interlocutors’ intention and social characteristics in a dynamic context, with a view to investigating the generation mechanism of the “V+*Ta*+XP” construction, based on the hypothesis that the virtual reference *Ta* comes from the abbreviation of *Ta x de*.

## 2 Theoretical background

This study adopts a cognitive-sociolinguistic framework, integrating construction grammar, politeness theory, and sociolinguistic variation to analyze the formation of the “V+Ta+XP” construction. Cognitive sociolinguistics examines language variation through the lens of social and cognitive constraints, emphasizing how grammar emerges from social communication needs and cognitive styles (Geeraerts et al., 2010; Hollmann, 2013; Li, 2025).

### 2.1 Construction grammar

Following Goldberg (1995), a construction is defined as a form-meaning pair where neither form nor meaning is fully predictable from its components. Ditransitive constructions, for example, encode transfer events (“X CAUSE Y TO RECEIVE Z”), with verbs like “give” aligning seamlessly with this structure. Constructional coercion occurs when verb semantics and constructional semantics mismatch. For instance, in “John sneezed the foam off the cappuccino,” the verb “sneeze” is coerced into the caused-motion construction. Shi (2012) extends coercion to include formal changes, such as Beijing dialect’s “er” sound assimilation: *guan* (can)+*er* → *guar* (can). In this study, the “V+Ta+XP” construction functions as a non-referential expressive marker that encodes emotional salience—including emphasis, catharsis, or jocularity—beyond propositional content. As such, the “V+Ta+XP” construction can be treated as a form-meaning pair where emotional meaning transcends the sum of its parts. The transition from “V+Ta x de+XP” to “V+Ta+XP” exemplifies formal coercion, driven by socio-cognitive factors. Since the meaning of “V+*T*a *x de*+XP” is the sum of every single part; thus, we call it “structure” to differentiate itself from “V+*Ta*+XP” construction.

### 2.2 Politeness

Politeness, as defined by Brown and Levinson (1987), is essential for maintaining social order and enabling cooperation. It operates through “face-work,” which Goffman (1955, 1956) describes as managing the social image individuals project during interactions. “Face” is the positive social value a person claims, and its preservation is a universal social necessity. In Chinese society, “face” is particularly delicate, serving as a social ideology that legitimizes status and regulates interaction (Lin, 1940; Stover, 1962). Acts like criticism or insults threaten “face,” prompting speakers to mitigate face-threatening effects. For instance, the expletive “Ta x de” may be replaced by the milder “Ta” to avoid social discomfort.

### 2.3 Variation

Language variation, as studied in sociolinguistics, refers to alternative linguistic forms expressing the same function, each carrying social or stylistic significance (Hudson, 1988; Labov, 1972). As such, a sociolinguistic variable is a linguistic element that is sensitive to a number of extralinguistic independent variables like social class, age, sex, and register (Chen and Liang, 2023). A simple example is that the second person singular has two forms in Mandarin, Ni “you” and Nin “you,” which are variations of the second person singular. This study focuses on how register and gender influence the “V+Ta+XP” construction.

The transition from “V+Ta x de+XP” to “V+Ta+XP” is driven by socio-cognitive factors, where social norms and cognitive emotions interact. For example, “da Ta x de yixiu majiang” (“play [TA] x de mahjong all night”) may become “da Ta yixiu majiang” to align with politeness principles. Here, “Ta x de” is coerced into “Ta,” retaining emotional expression while avoiding face-threatening elements. This process, termed “socio-cognitive coercion,” transforms a temporary linguistic variant into a solid construction.

## 3 Data and methodology

### 3.1 Corpus

This paper makes use of data from the searchable Internet version of the CCL Chinese Corpora created and managed by Peking University, and from BCC corpus by Beijing Language and Culture University.

The CCL corpora were established in 2003 and include data of about 716,000,000 tokens of characters in Modern Chinese and Comtemporary Chinese. The source distribution is shown in Table 1.

**Table 1:** The source distribution of CCL.

Corpus	Sources	Proportion(%)
The modern Chinese corpus	Drama	7.85
	Literature	92.15
The contemporary Chinese corpus	Spoken Language	0.22
	Historical Biography	0.76
	Practical Articles	4.18
	Newspaper	72.73
	Literature	7.38
	Film and Television	1.85
	Comic Sketches	0.30

The BCC corpus is about 14 billion words, of which 2 billion, or 14%, are in Newspapers; 3 billion, or 22%, each are in Literature, Microblog and Science and Technology; 1 billion, or 7%, are in General; and 2 billion, or 14%, are in Ancient Chinese. The distribution is relatively even, which can show the language life of today’s society in a comprehensive way. The source distribution is shown in Table 2.

**Table 2:** The source distribution of BCC.

Sources	Frequency	Proportion(%)
Newspaper	2 billion	14
Literature	3 billion	22
Microblog	3 billion	22
Science and Technology	3 billion	22
General	1 billion	7
Ancient Chinese	2 billion	14

These two corpora are both spoken language, which is consistent with the special spoken Mandarin construction “V+*Ta*+XP,” so they are applicable for the data collection of this study. It should be noted that in the initial research process, we only adopted the CCL corpus and collected 160 “V+*Ta*+XP” constructions and 97 “V+*Ta x de*+XP” structures, which is not conducive to the research due to the small amount of data. Then, we extend the corpus collection to the BCC corpus, which includes four major domains of modern Chinese. Thus, this paper combines the corpus from the CCL corpus and the BCC corpus to conduct a more comprehensive and powerful study.

### 3.2 Data extraction

This study retrieved about 1.46 million entries containing *Ta* in the CCL corpus. By reading them one by one, 906 entries matching the configuration of “V+*Ta*+XP” were selected and manually screened again. Each corpus sample was reviewed, deleted, and merged to finally obtain 160 corpora of the subjects of this study.

In addition, in order to collect the structure “V+*Ta x de*+XP,” this paper searches the possible forms of the expletive *Ta x de* in CCL corpus: “他妈的”*Ta ma de*, “他娘的” *Ta niang de*, “他奶奶的” *Ta nainai de*, “他狗日的” *Ta gouri de*, etc. Among them, there is only one case of *Ta nainai de*, *Ta gouri de* inserted into the verb-object (complement) construction, which cannot fully reflect the register characteristics of their distribution. Therefore, this paper focuses on the data collection of “V+*Ta ma de*+XP,” “V+*Ta niang de*+XP.”

A search for the phrase *Ta ma de* in the CCL corpus retrieved a total of 1715 utterances. It was found that the syntactic position of the expletive phrase *Ta ma de* is highly flexible and can be inserted between a variety of grammatical constituents; it can appear outside the sentence, between subject and predicate, between the verb and the object, between the verb and the complement, at the beginning of the clause, etc. 59 sentence patterns matching the “V+*Ta ma de*+XP” structure were manually selected. The phrase *Ta niang de* was extracted in the same fashion. Of the 336 corpus retrieved, 38 were manually checked out to match the “V+*Ta niang de*+XP” structure. Thus, there were 97 “V+*Ta x de*+XP” in total in CCL. The specific distribution is shown in Table 3.

**Table 3:** The specific distribution of *Ta x de* in CCL.

Ta x de	Total frequency	“V+Ta x de+XP”	Proportion (%)
Ta ma de	1715	59	3.44
Ta niang de	336	38	11.31
Total	2051	97	4.73

The same retrieval was carried out in the BCC corpus. Since the BCC corpus has the retrieval function of general format, this paper took *V Ta* as the retrieval character, and finally obtained 447 “V+*Ta*+XP” constructions.

The phrase *Ta ma de* was searched in BCC and 2,405 corpora were retrieved. A total of 270 “V+*Ta ma de*+XP” were manually extracted. A search for *Ta niang de* yielded 269 corpora, of which 35 were manually checked out in line with the “V+*Ta niang de*+XP” structure. Totally, there are 305 “V+*Ta x de*+XP” in BCC. The specific distribution of “V+*Ta x de*+XP” in BCC is shown in Table 4.

**Table 4:** The specific distribution of *Ta x de* in BCC.

Ta x de	Total frequency	“V+Ta x de+XP”	Proportion (%)
Ta ma de	2,405	270	11.23
Ta niang de	269	35	14.13
total	2,674	305	11.41

In total, there are 607 “V+*Ta* +XP” constructions, with 160 in CCL and 447 in BCC, and there are 402 “V+*Ta x de*+XP” structures, with 97 in CCL and 305 in BCC.

### 3.3 Data analysis

The primary objective of the data analysis was to test the hypothesis that the “V+*Ta*+XP” construction origins from the “V+*Ta x de*+XP” structure, and explore the mechanism of the formation of the “V+*Ta*+XP” construction.

Based on the data collected, firstly, we analyze the syntactic features, especially the XP characteristics of the “V+*Ta x de*+XP” structure and the “V+*Ta*+XP” construction. Secondly, we analysis the pragmatic functions of the two. Thirdly, we make a statistic analysis of the register distribution and gender characteristics of communicators of the “V+*Ta x de*+XP” structure and the “V+*Ta*+XP” construction, respectively.

By the comparative analysis of the “V+*Ta x de*+XP” structure and the “V+*Ta*+XP” construction, we can notice the similarities and differences between the two, further proposing the formation mechanism of the “V+*Ta*+XP” construction.

## 4 Results

This section presents the findings of the corpus analysis, focusing on syntactic configurations, pragmatic functions, register distribution, and gender characteristics of the “V+Ta+XP” construction and its precursor “V+Ta x de+XP” structure.

### 4.1 Syntactical features of the XP component

The XP slot in the “V+Ta+XP” construction and the “V+Ta x de+XP” structure exhibits two primary configurations: (1) ge+(with the classifier “ge”) and (2) ge- (without “ge”). As shown in Tables 5, 6, the ge+type demonstrates complete parallelism across both structures, encompassing quantitative phrase, idiom, adverb, adjective, and noun. For instance, Shui Tamade ge yitian (“Sleep Tamade ge one day”) and its abbreviated form “Shui Ta ge yitian” share identical syntactic structures and propositional meanings.

**Table 5:** The internal configurations of the two structures with XP *ge+.*

XP component	An example of “V+Ta x de+XP”	An example of “V+Ta+XP”
Ge+quantitative phrase	Shui Tamade ge yitian “Sleep Tamade ge one day”	Shui Ta ge yitian
Ge+idiom	Da Tamade ge luohualiushui “Knock Tamade ge out of me”	Da Ta ge luohualiushu
Ge+adverb	He Tama ge gou “Drink Tama ge enough”	He Ta ge gou
Ge+adjective	He Tama ge tongkuai “Drink Tama ge delightedly”	He Ta ge tongkuai
Ge+noun	Gao Tama ge lanxiaoxuntong “Get Tama ge a bad school”	Gao Ta ge lanxiaoxuntong

**Table 6:** The internal configurations of the two structures with XP *ge-.*

XP component	An example of “V+Ta x de+XP”	An example of “V+Ta+XP”
Bare noun	Shui Ta made jiao “sleep Ta made”	/
“Shenmo”+bare noun	Chi Ta ma shenmo shuiguo “eat Ta ma what fruit”	/
Quantitative phrase	He Ta niangde yidun “drink Ta niangde a time”	He Ta yidun
Quantitative phrase+noun1	He Ta niangde jibei qinggongjiu “drink Ta niangde a couple of cheers”	He Ta jibei qinggongjiu
Quantitative phrase+noun2	He Ta niangde yigexingqi zhongyao “drink Ta niangde a week of Chinese medicine”	He Ta yigexingqi zhongyao
Temporal noun	Huo Ta ma daban beizi “live Ta ma most of his life”	Huo Ta daban beizi

The ge-type, however, diverges in specific contexts. While both structures permit quantitative phrase, quantitative phrase+noun1, quantitative phrase+noun2, and temporal noun, the “V+Ta x de+XP” structure uniquely accommodates bare noun (e.g., shui Ta ma de jiao, “sleep Ta made”) and shenmo (“what”)-modified nouns (e.g., chi Ta ma shenmo shuiguo, “eat Ta ma what fruit”). These configurations lack direct equivalents in the “V+Ta+XP” construction unless augmented with ge or quantifiers (e.g., shui Ta ge jiao).

It should be noted that for “quantitative phrase+noun1,” the quantitative phrase modifies the noun, e.g., in “He Ta niangde jibei qinggongjiu” (“drink Ta niangde a cup of wine”), the quantitative phrase “jibei” (“a cup of”) modifies the noun “qinggongjiu” (“wine”); for “quantitative phrase+noun2,” the quantitative phrase modifies the preceding verb, e.g., in “He Ta niangde yigexingqi zhongyao” (“drink Ta niangde a week of Chinese medicine”), the quantitative phrase “yigexingqi” (“a week of”) modifies the verb “He” (“drink”).

### 4.2 Pragmatic functions

It has been shown that swearing words like “Ta x de” have the pragmatic functions of swearing, evaluation (Chou and Zhang, 2005, p. 25), venting emotions, and expressing intimacy (Leech, 1983; Daly et al., 2004; Bernal, 2008; Haugh and Bousfield, 2012). “Lack of politeness” sometimes has the effect of “establishing or maintaining a bond of familiarity” and “creating or affirming solidarity” (Leech, 1983, p. 144).

The examination of the utterances containing the “V+*Ta*+XP” construction based on the data has found that the construction has the function of evaluation, expression of emotions toward others, and expression of the self-emotion. The “evaluation function” in this paper refers to an opinion about a person or an event; The “expression of emotions toward others” refers to the speaker’s encouragement, instruction, expectation, exhortation, complaint, etc. to others; The “expression of the self-emotion” is an internal mental activity, directed to the self, which indicates an expression of emotion about the self or the self-related events.

Both structures serve expressive functions, yet they diverge in emotional intensity. The “V+Ta x de+XP” structure carries strong vulgarity (e.g., he Ta ma de ge tongkuai, “have a fucking drink”), aligning with swearing, evaluation, and emotional venting (Bernal, 2008; Haugh and Bousfield, 2012). In contrast, the “V+Ta+XP” construction mitigates vulgarity while retaining emotional emphasis (e.g., he Ta ge tongkuai, “have a [Ta] drink”). Repetition or adverbial intensification (e.g., zhenzheng de, “truly”) amplifies emotional expression, as shown below:

(4) 尼克: 咱们喝他个痛快!

Nick: *Zan men he Ta ge tongkuai*!

Nick: Let us have a [Ta] good drink.

比尔:咱们喝他个真正的一醉方休!

Bill: *Zan men he Ta ge zhenzheng de yizuifangxiu!*

Bill: Well, let us get [*Ta*] really drunk. (Hemingway/3 days of wind).

### 4.3 Register distribution

The register distribution of the “V+Ta x de+XP” construction in the CCL corpus demonstrates marked text-type specificity. Of the 97 attested instances, no occurrences were found in Historical Biography, Practical Articles, or Comic Sketches. The predominant concentration resides in Literature (74 instances, 76.29%), followed by Newspaper (15, 15.46%), Film and Television (6, 6.19%), and minimal attestations in Internet Corpus and Drama (1 each, 1.03%; see Table 7).

**Table 7:** Register distribution of “V+*Ta x de*+ XP” and “V+*Ta*+XP” in CCL.

Text type	V+Ta x de+XP	proportion(%)	V+Ta+XP	Proportion (%)
Historical Biography	/	/	1	0.62
Practical Articles	/	/	4	2.5
Newspaper	15	15.46	49	30.63
Literature	74	76.29	97	60.62
Film and Television	6	6.19	8	5
Comic Sketches	/	/	1	0.62
Internet Corpus	1	1.03	/	/
Drama	1	1.03	/	/
Total	97	100	160	100

In contrast, the “V+Ta+XP” construction (*N* = 160) exhibits broader register adaptability, though still skewed toward Literature (97, 60.62%) and Newspaper (49, 30.63%). Secondary distributions occur in Film and Television (8, 5%), Practical Articles (4, 2.5%), with marginal instances in Historical Biography and Comic Sketches (1 each, 0.62%; Table 7).

The BCC data reveals divergent patterns. For “V+Ta x de+XP” (*N* = 305), Literature remains dominant (187, 61.31%), followed by Microblog (86, 28.2%), Newspaper (21, 6.89%), and Science & Technology (11, 3.61%; Table 8).

**Table 8:** Register distribution of “V+*Ta x de*+ XP” and “V+*Ta*+XP” in BCC.

Text type	“V+Ta X de+XP”	proportion(%)	“V+Ta+XP”	Proportion (%)
Literature	187	61.31	81	18.16
Newspaper	21	6.89	116	26.01
Microblog	86	28.2	202	45.29
Science&Technology	11	3.61	48	10.76
Total	305	100	447	100

The “V+Ta+XP” construction (*N* = 462), however, shows a distinct register profile: Microblog leads with 202 instances (45.29%), followed by Newspaper (116, 26.01%), Literature (81, 18.16%), and Science & Technology (48, 10.76%). Notably, the verb shui (“sleep”) accounts for 107 cases (53%) in Microblog, suggesting a pragmatic association with quotidian exclamations.

To determine whether the register distributions of “V+Ta x de+XP” and “V+Ta+XP” differed significantly, a chi-square test of independence was conducted (Table 8). The result revealed a statistically significant association between construction type and register distribution: *χ*^2^ (3, *N* = 752) = 156.59, *p* < 0.001, Cramér’s V = 0.456 (indicating a moderate to large effect size). Analysis of standardized residuals identified key distributional contrasts: “V+Ta x de+XP” was significantly overrepresented in Literature (residual = +7.52). Its distribution was heavily skewed toward registers associated with orality—predominatintly occurring in literary dialog mirroring informal spoken language (61.31%) with secondary presence in Microblog (28.2%)—but showed marginal occurrence in formal registers like Newspaper (6.89%) and Science & Technology (3.61%).

Conversely, “V+Ta+XP” demonstrated register flexibility. It was overrepresented not only in Microblog (residual = +2.35) but also in Newspaper (residual = +3.83), dominating the infromal Microblog register (45.29%) while maintaining substantial frequency in formal registers like Newspaper (26.01%) and Science & Technology (10.76%).

This functional divergence is exemplified by the contrast between the vulgar expression *kan ta ma ge gou* (“see [Ta] fucking enough”) in the informal Microblog register and its de-vulgarized counterpart *kan ta ge gou* (“see [Ta] enough”) in Science & Technology register.

(5) *rang ni kan ta ma ge gou*! (Microblog)

Let you see (sth) fucking enough.

(6) *yi ding kan ta ge gou*! (Science & Technology)

You should see (sth) enough.

Crucially, the analysis confirms that “V+Ta x de+XP” exhibits limited productivity in formal registers, whereas “V+Ta+XP” displays cross-register functionality, albeit with frequency gradations tied to text-type norms.

### 4.4 Gender characteristics of communicators

The gender distribution patterns of the “V+Ta x de+XP” and “V+Ta+XP” constructions reveal marked sociolinguistic asymmetries. After excluding gender-ambiguous Microblog data (86 instances for “V+Ta x de+XP”; 202 for “V+Ta+XP”), the analyzable dataset comprised 316 valid cases for “V+Ta x de+XP” and 405 cases for “V+Ta+XP.” Gender categorization distinguished monologic contexts (speaker gender explicitly marked, e.g., “Male,” “Female”) from dyadic interactions (speaker-addressee pairs, e.g., “Male–Male,” “Female–Male”).

Quantitative results highlight persistent male dominance across both constructions. For “V+Ta x de+XP” (*N* = 316), male monologic use accounted for 35.76% (113 cases), while male–male constituted 38.29% (121 cases), totaling 74.05% of all instances. Female participation remained marginal: monologic use represented 2.85% (9 cases), and female–female a mere 1.27% (4 cases). Cross-gender interactions exhibited asymmetry, with male→female exchanges (7.28%, 23 cases) surpassing female→male instances (4.75%, 15 cases).

The “V+Ta+XP” construction (*N* = 405) displayed similar yet moderated gender disparities. Male monologic use (30.12%, 122 cases) and male–male (35.06%, 142 cases) collectively represented 65.18% of cases. Female agency was even more constrained: monologic use dropped to 0.7% (3 cases), and female–female marginally increased to 1.73% (7 cases). Cross-gender interactions followed the same asymmetry, with male→female (4.69%, 19 cases) exceeding female→male (2.96%, 12 cases).

To statistically evaluate the association between construction type and gender distribution, a chi-squared test of independence was performed on the data in Table 9. The null hypothesis posited no relationship between the two variables (i.e., gender distribution is independent of construction type). The test yielded a significant result: *χ*^2^ (6, *N* = 721) = 32.40, *p* < 0.001. This indicates a statistically significant association between the construction type and gender distribution patterns.

**Table 9:** The gender distribution of communicators for “V+*Ta x de*+XP” and “V+*Ta*+XP” In CCL and BCC.

Gender	“V+Ta X de+XP”	Proportion(%)	“V+Ta+XP”	Proportion (%)
Male	113	35.76	122	30.12
Female	9	2.85	3	0.7
Male–male	121	38.29	142	35.06
Male–female	23	7.28	19	4.69
Female–female	4	1.27	7	1.73
Female–male	15	4.75	12	2.96
Unknown gender	31	9.81	100	24.69
Total	316	100	405	100

Analysis of standardized residuals identified the most significant contributor to this association: the disproportionately high frequency of “unknown gender” instances in the “V+Ta+XP” construction (observed = 100, expected = 73.59; residual = +3.08, *p* < 0.05). Additionally, while the observed frequency of female monologic use in “V+Ta+XP” was lower than expected (observed = 3, expected = 6.74; residual = −1.44, *p* > 0.05), this difference did not reach conventional levels of statistical significance.

Regarding test assumptions, 12 out of 14 cells (85.7%) had expected frequencies greater than 5. Although two cells had expected frequencies below 5 (minimum = 4.82), the proportion of such cells (14.3%) is within commonly accepted limits for chi-square validity.

Gender distribution reveals notable sociolinguistic constraints (Table 9). Male speakers dominate both constructions (35.76% for “V+Ta x de+XP”; 30.12% for “V+Ta+XP”), particularly in male–male interactions (38.29% vs. 35.06%). Female usage is minimal (2.85% vs. 0.7%), likely due to face-preservation norms discouraging vulgarity among female speakers (Brown and Levinson, 1987). Critically, the “V+Ta+XP” construction demonstrates significantly broader sociolinguistic distribution, evidenced by its higher proportion of unknown gender cases (24.69% vs. 9.81%). This pattern suggests “V+Ta+XP” functions as a more socially neutral variant, frequently occurring in gender-unspecified contexts where sociolinguistic markedness is reduced.

## 5 Discussion

### 5.1 Syntactic and pragmatic parallelism

The structural parallelism between “V+Ta x de+XP” and “V+Ta+XP” supports the hypothesis that “Ta” originates from “Ta x de” abbreviation. Both constructions license ge+configurations (e.g., ge+quantitative phrase), while differences in ge-types (e.g., bare noun) underscore the necessity of quantifier insertion for grammaticality.

Pragmatically, the “V+Ta+XP” construction serves as a de-vulgarized variant, attenuating emotional intensity while retaining evaluative and expressive functions.

Let us look at a group of examples and compare the following three groups of sentence patterns:

**Table:** 

**A:vulgarization** V+Ta x de+XP	**B:de vulgarization** V+Ta+XP	**C:neutrality** V+XP
(7) *he ta ma de ge tongkuai* have a fucking drink	*he ta ge tongkuai* have a [Ta] drink	*he ge tongkuai* have a drink
(8) *gan ta niang de yibeizi* work for a fucking lifetime	*gan ta yibeizi* work for a [Ta] lifetime	*gan yibeizi* work for a lifetime
(9) *chi ta ma de yidun* have a fucking meal	*chi ta yidun* have a [Ta] meal	*chi yidun* have a meal

The above three groups of sentences exhibit the following characteristics: (1) In terms of syntactic information, there is no difference between the three groups of sentences, all of them are verb-object or verb-complement structures. (2) In terms of semantic information, they all express the semantic meaning of category C. (3) In terms of pragmatic information, category C expresses neutral emotion, except when the insertion of *ge* expresses a certain emotional meaning in *he ge tongkuai*, category B expresses more emotional meaning due to the insertion of *Ta*, and the insertion of *x de* (e.g.: *ma de*; *niang de*) in category C makes the emotional meaning more abundant. Compared with sentence pattern A, sentence B pattern is a process of de-vulgarization.

The “V+*Ta*+XP” construction expresses weaker emotions than the “V+*Ta x de*+XP” structure. Sometimes the speaker or writer will use the “V+*Ta*+XP” construction twice (as in example (10)), or add an intensifying adverbial modifier to the XP component (as in example (11)) to strengthen the emotional expression.

(10) 好!好!干他个痛快!干他个痛快!

*Hao! Hao! Gan Ta ge tongkuai! Gan Ta ge tongkuai!*

OK! OK! Have a [Ta] good drink! Have a [Ta] good drink!

(contemporary\literature\mainland writer\Qu Bo Linhai snow field. Txt).

(11) 尼克: 咱们喝他个痛快!

Nick: *Zan men he Ta ge tongkuai*!

Nick: Let us have a [Ta] good drink.

比尔:咱们喝他个真正的一醉方休!

Bill: *Zan men he Ta ge zhenzheng de yizuifangxiu!*

Bill: Well, let us get [*Ta*] really drunk. (Hemingway/three days of wind).

In (10), the speaker used *gan Ta ge tongkuai* “have a [Ta] good drink” twice in a row, which conveys a high-level emotional state. According to Potts (2007), if a speaker repeatedly uses an expressive item, the effect is generally to strengthen the emotional content, rather than to be redundant, which basically conforms to the Quantity Principle in Iconicity proposed by Haiman (1985, p. 147), which means that the formal complexity should correspond to the conceptual complexity.

In (11), when “Nick” said *he Ta ge tongkuai* “have a [Ta] good drink” to express a medium amount of emotional meaning, “Bill” responded with *he Ta ge zhen zheng de yizuifangxiu* “get [*Ta*] really drunk,” in which *zhen zheng de* strengthened the result of getting drunk, thus raising the emotional potential to a high level.

When the speaker is extremely full of emotion, the “V+*Ta x de*+XP” structure is used, e.g.,

(12) 今天我要使劲熬个夜，明天狠狠地狠狠地睡他妈一个懒觉!(微博)

*Wo yao shi jin ao ge ye, ming tian henhende henhende shui Ta ma yige lanjiao*.

I’m going to stay up hard today and *sleep* [Ta] fucking hard tomorrow! (Microblog).

The use of the degree adverb *henhende* “hard” here indicates that the blogger’s desire to sleep is extremely strong and the emotions run high, hence the “V+*Ta x de*+XP” structure and the exclamation mark are used to reinforce this emotion.

In short, both the “V+*Ta*+XP” construction and the “V+*Ta x de*+XP” structure can convey special emotional meaning beyond the expression of the propositional meaning, with the former containing an intermediate level of emotional meaning and the latter containing a high level of emotional meaning. In addition, the “V+*Ta*+XP” construction does not have the curse meaning contained in the “V+*Ta x de*+XP” structure, and it is the result of the “de vulgarization” of the latter, which means that when the “V+*Ta x de*+XP” structure is selected as the language potential for emotional expressions, it may undergo variant behavior due to socio-cognitive factors, and the “V+*Ta*+XP” construction will become the final linguistic expression.

It should be noted that “V+Ta+XP” construction operates within a constructional network, comprising four core patterns that collectively map affective meaning onto structural formality. Prototypically, this system manifests: (1) V+de+XP (e.g., 吵得 (个)不亦乐乎*chao de (ge) buyilehu*, “make de (ge) an uproar”), where “de” encodes realized results of completed actions, occupying the formal register pole with neutral expressivity; (2) V+ge+XP (e.g., 闹个人仰马翻 *nao ge renyangmafan*, “stir up ge utter chaos”), where the classifier “ge” quantizes unbounded events while optionally marking prospective actions, exhibiting neutral-to-moderate affect across registers; (3) V+Ta+XP encodes prospective actions (unrealized events) and speaker attitude, representing a semiformal, de-vulgarized variant with moderated emotional intensity; (4) V+Ta x de+XP deploys the vulgarized “x de” to convey high-intensity affect, anchoring the informal/vulgar pole. This continuum is governed by affective gradation progressing from neutral through moderate to high intensity, and register constraints spanning formal, semiformal, and informal domains.

While acknowledging this systemic relationship, the present analysis specifically examines the socio-cognitive coercion mechanism underlying Ta’s emergence from its vulgar precursor V+Ta x de+XP. Delineating functional boundaries across this continuum—particularly aspectual interactions and register-sensitive constraints—constitutes essential future work (Section 6.3).

### 5.2 Contextual constraints and socio-cognitive coercion

Register and gender analyses reveal how socio-cognitive factors drive variation. The “V+Ta x de+XP” structure is restricted to informal contexts due to its face-threatening vulgarity, whereas the “V+Ta+XP” construction thrives in formal registers by balancing emotional expression with politeness (Goffman, 1955).

The gender skew aligns with face-management strategies (Brown and Levinson, 1987) and gendered politeness norms. Male overrepresentation, particularly in male–male dyads, suggests greater tolerance for assertive/face-threatening acts within masculine discourse communities. Conversely, the near-erasure of female speakers—both monologic (≤2.85%) and dyadic (≤4.75%)—reflects social sanctions against women’s use of confrontational/vulgar language, consistent with cross-cultural observations of gendered pragmatic constraints (Eckert and McConnell-Ginet, 1992). Notably, the “V+Ta+XP” construction demonstrates broader gender adaptability compared to “V+Ta x de+XP,” likely due to its functional neutralization as a socially safer variant.

Brown and Levinson (1987) proposed that when the speaker criticizes, despises and insults the receiver, it will pose a threat to the receiver’s positive face. Although jocular mockery (Haugh, 2010, 2011; Haugh and Bousfield, 2012) and jocular abuse (Hay, 2002; Haugh, 2009, p.77–78) were found to arise in casual interactions between friends and family members to maintain intimacy, the insertion of the curse *Ta x de* is as often as not insulting in most other occasions, it can cause the face violation of the addressee, which makes people try to avoid the use of this sentence pattern as much as possible in normal discourse situations, so as to preserve their social face and mitigate the threat of face loss. Specifically, the “V+*Ta x de*+XP” structure with the insertion of abusive language “*x de”* is inappropriate and not in line with the social pragmatic norms in some discourse environments due to the social distance and relative power relations between the communicators and face want, which may cause the de-vulgarization of “V+*Ta x de*+XP” into “V+Ta+XP.”

**Figure 1:**
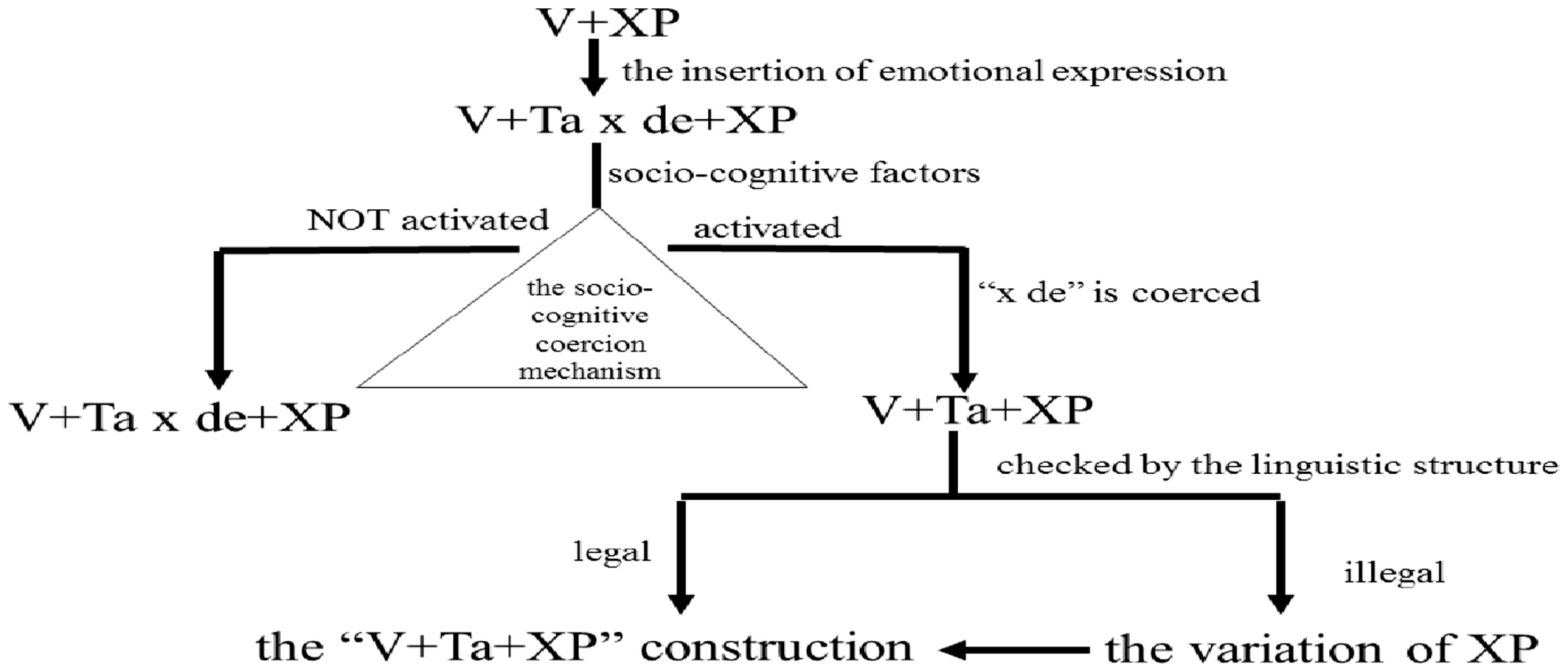
The “socio-cognitive coercion mechanism” of the formation of the “V+*Ta*+XP” construction.

The proposed socio-cognitive coercion mechanism (Figure 1) explains this transition. When social norms (e.g., register formality, gender expectations) conflict with the use of “Ta x de,” the abusive component “x de” is coerced, yielding “V+Ta+XP.” Grammatical verification ensures structural legitimacy: XP components lacking quantifiers (e.g., bare noun) require “ge” insertion (e.g., shui Ta ge jiao), aligning with Mandarin’s syntactic rules.

As shown in Figure 1, the verb-object construction “V+XP” is constructed when a propositional event needs to be expressed (e.g., “da (playing) mahjong”), and when special emotional expressions are needed, they are expressed with the help of interjectional morphology, such as *Ta ma de*, *Ta niang de*, *Ta nainai de*, *Ta gou ri de*, forming “V+*Ta x de*+XP.” Once this structure is used in social communication, it will be licensed by the social factors, such as face, the communicator’s gender, register, the occasion of speaking. If these factors are matched with the use of abusive words, i.e., they meet the needs of social communication and do not affect the communicator’s face, the “socio-cognitive coercion” mechanism is not activated, the speaker directly outputs “V+*Ta x de*+XP”; when the socio-cognitive factors do not match the use of abusive words, in order to maintain the face of both communicators, the “socio-cognitive coercion” mechanism can be activated to exert a repressive effect on the abusive words *Ta x de* under the effect of social and moral regulations. Three conditions must be met for successful coercion:

1. Conform to the structure allowed by the existing Chinese sentence patterns;
2. Helps to highlight the transmission of emotions;
3. “De-vulgarization” of the form.

Specifically, *Ta x de* in “V+*Ta x de*+XP” can be coerced in seven ways: ① the first morpheme *Ta* in *Ta x de* is coerced. In this case, the expletive *x de* is still in the structure and does not meet the condition (3) above, so the coercion is invalid. ② The second morpheme *x* in *Ta x de* is coerced. At this point, the remaining structure “V+*Ta de*+XP” (e.g., *da ta de yixiu mahjong*), where *de* is more like an attribute mark, and *Ta* and *mahjong* are affiliated, cannot express special emotional meaning, i.e., they do not meet the above condition (2), so the coercion is still invalid. ③ The third morpheme *de* in *Ta x de* is coerced, and the abusive component *x* still exists, which does not meet condition (3), so the coercion is invalid. ④ The first two morphemes *Ta x* in *Ta x de* are coerced, leaving “V+*de*+XP,” which is not always grammatical and does not comply with the conditions (1), so this suppression is not desirable. ⑤ The first morpheme *Ta* in *Ta x de* is coerced together with the third morpheme *de*, leaving “V+*x*+XP,” which neither achieves the effect of “de-vulgarization” nor conforms to the grammatical structure, i.e., it does not comply with conditions (1) and (3), so this coercion is not desirable. ⑥ The second morpheme *x* in *Ta x de* is coerced together with the third morpheme *de*, leaving “V+*Ta*+XP,” which not only achieves the effect of “de-vulgarization,” but conforms to the configuration of typical double object construction and expresses a certain emotional meaning, that is to say, the above three conditions are satisfied at the same time, so this coercion is effective. ⑦ The whole phrase *Ta x de* is coerced into a “V+XP” single-object structure, which cannot convey emotional meaning, that is, it does not meet the condition (2). That said, only the sixth of the seven possible coercion methods satisfies all these three conditions at the same time and is the only valid coercing method.

After *x de* in “V+*Ta x de*+XP” being coerced by socio-cognitive factors, the formed “V+*Ta*+XP” structure needs to be checked by the linguistic structure to see if it is grammatical. Only “V+*Ta*+XP” with grammatical legitimacy can enter the linguistic category of “V+*Ta*+XP” construction and become a legal independent linguistic item stored in the mental lexicon. For “V+*Ta*+XP” that fails the verification mechanism, it will not form an independent linguistic item, and the XP component needs further variation. Specifically, when XP contains quantitative components, it is a legal structure, which is directly output to form the “V+*Ta*+XP” construction; When XP is a bare noun or “*shenmo*+bare noun,” the “V+*Ta*+XP” is not grammatical and XP needs to be slightly changed: it is made legal by adding (number) quantifiers, e.g., *ge*. For example, it is illegal to say *kai Ta hui* “have [Ta] meeting”; after adding (number) quantifiers, *kai Ta ge hui* becomes a legal construction.

The “V+*Ta*+XP” structure formed out of the “socio-cognitive coercion” in a given communication is gradually solidified from a temporary variant form into an independent linguistic item. That is to say, *Ta x de* inserted into the verb object (complement) structure “V+XP” is compressed into *Ta* due to the constraints of socio-cognitive factors. The formed “V+*Ta*+XP” structure becomes valid before the linguistic verification mechanism. This abbreviation is a temporary linguistic variant in the current context, but the temporary variant gradually precipitates into an independent structure after normalization, and the internal structure is no longer constitutive. This is the reason why the “V+*Ta*+XP” construction has “no place” in the syntactic structure. Although before the abbreviated variant, *da Ta x de yixiu mahjong* can be divided into the combination of *da yixiu mahjong* and *Ta x de*, in which *Ta x de* can be moved from the middle position to the front *Ta x de, da yixiu mahjong*, or the back *da yixiu mahjong, Ta x de*. However, the variant *da Ta yixiu mahjong* cannot be converted into *Ta*, *da yixiu mahjong* and *da yixiu mahjong, Ta*. That is to say, *Ta*, the abbreviated form of *Ta x de*, and the verb object (complement) structure “V+XP” have “fused” into an indivisible linguistic structure, which is why it will be difficult to trace back the source of the variant form *da Ta yixiu mahjong*.

## 6 Conclusion

### 6.1 Summary of key findings

This study investigated the formation mechanism of the Mandarin “V+Ta+XP” construction, focusing on its syntactic, pragmatic, socio-cognitive contextual similarities with and divergence from the vulgar precursor “V+Ta x de+XP.” Through a corpus-based analysis of 607 “V+Ta+XP” constructions and 402 “V+Ta x de+XP” structures, three key findings emerge:

First, structural and functional parallelism: The “V+Ta+XP” construction retains syntactic configurations (e.g., ge+and ge-types) and pragmatic functions (evaluation, emotional expression) of its precursor “V+Ta x de+XP,” while systematically suppressing vulgar components “x de.” This supports the hypothesis that “Ta” originates from the abbreviation of “Ta x de,” functioning as a non-referential insert to mitigate face-threatening elements.

Second, register and gender constraints: The “V+Ta x de+XP” structure is restricted to informal registers (e.g., Microblog) and male-dominated interactions, reflecting its socially marked vulgarity. In contrast, the “V+Ta+XP” construction demonstrates broader acceptability in formal contexts (e.g., Newspaper, Science & Technology) and across genders, aligning with politeness norms (Brown and Levinson, 1987).

Third, socio-cognitive coercion mechanism: The transition from “V+Ta x de+XP” to “V+Ta+XP” is driven by a dynamic interplay of social norms (e.g., face preservation, register formality) and cognitive-emotional needs. Grammatical verification ensures structural legitimacy, requiring quantifier insertion (e.g., “ge”) for bare nouns, thereby solidifying “V+Ta+XP” as an independent construction.

### 6.2 Theoretical and practical implications

By integrating socio-cognitive factors (e.g., face preservation, gendered politeness norms) into the framework of constructional coercion, this research bridges a critical gap in Goldberg’s construction grammar, which traditionally prioritizes cognitive-semantic motivations over sociolinguistic constraints. The proposed socio-cognitive coercion mechanism demonstrates that grammaticalization is not merely a product of structural reanalysis but a negotiated outcome of social norms (e.g., register appropriateness) and cognitive-emotional needs (e.g., expressive salience). This aligns with recent calls for a “social turn” in cognitive linguistics (Hollmann, 2013; Geeraerts et al., 2010) and enriches coercion theory by formalizing how extralinguistic factors systematically suppress or license structural variants.

Practically, the findings offer significant pedagogical insights for Mandarin learners, highlighting the contextual sensitivity of emotional expressions and the importance of register-appropriate language use. Specifically, they underscore the necessity of register-sensitive instruction: while the “V+Ta+XP” construction (e.g., 喝他个痛快) may be acceptable in informal narratives, its use in formal writing (e.g., academic texts) remains marked. Explicit teaching of the construction’s sociopragmatic boundaries—particularly its gendered usage skew—can mitigate pragmatic failures in cross-cultural communication. Key pedagogical applications for Teaching Mandarin as a Second Language include: (1) Utilizing corpus examples to contrast the vulgar (“V+Ta x de+XP”) and de-vulgarized (“V+Ta+XP”) forms; (2) Designing register-based exercises (e.g., comparing role-plays and academic writing tasks); (3) Addressing the observed gender asymmetry in usage patterns; (4) Demonstrating the construction’s role in emotional scaling (e.g., comparing *He santian jiu* (“drink for three days”) with *He Ta santian jiu* (“drink Ta for three days”), *He ge santian jiu* (“drink ge for three days”), and *He Tamade santian jiu* (“drink Ta made for three days”)). These strategies equip learners to effectively balance expressiveness with sociopragmatic norms. In natural language processing, recognizing “V+Ta+XP” as a de-vulgarized construction with retained emotional salience can improve sentiment analysis systems. For instance, differentiating between neutral 喝三天酒 (“drink for three days”) and expressive 喝他三天酒 (“drink [Ta] for three days”) requires algorithms to account for socio-cognitive cues beyond syntactic patterns.

### 6.3 Limitations and future directions

While this study provides novel insights, three limitations necessitate future research. First, while this study focused on register and gender as key social variables, future research should incorporate additional sociolinguistic dimensions—particularly social distance (e.g., intimacy between interlocutors) and power dynamics (e.g., hierarchical relationships in workplace or familial contexts)—to better capture the full spectrum of socio-cognitive constraints. For instance, the use of “V+Ta+XP” may vary significantly depending on whether speakers interact with close friends (low social distance) versus authority figures (high power asymmetry), potentially modulating the need for de-vulgarization. A more granular analysis of these factors could reveal how linguistic choices reflect and reinforce social hierarchies or solidarity in Mandarin communication. Simultaneously, building upon the established affective-register continuum of the “V+de/ge/Ta+XP” network (Section 5.1), future research would benefit from a systematic comparative analysis of “V+Ta+XP” with its adjacent constructions, V+de+XP and V+ge+XP. Such analysis should focus on key functional dimensions, particularly aspectual distinctions, quantification strategies, register sensitivity, and socio-cognitive constraints and motivations governing variant selection within speech communities. Second, the proposed coercion mechanism invites cross-linguistic validation. For instance, Japanese やつ (yatsu)-insertion (e.g., 飲んでやつ nonde yatsu “drink [yatsu]!”) and Korean 놈 (nom)-embedding (e.g., 먹을 놈 meogeul nom “eat [nom]!”) exhibit analogous de-vulgarization patterns worthy of comparative study. Third, psycholinguistic experiments (e.g., acceptability judgments, eye-tracking) could test whether native speakers process “V+Ta+XP” as a fused construction or a compositional variant. Such data would clarify the cognitive reality of socio-cognitive coercion and its role in online production.

In conclusion, the “V+Ta+XP” construction exemplifies how language evolves through the negotiation of social constraints and cognitive demands, offering a model for analyzing similar phenomena in other linguistic systems.

## Data Availability

The raw data supporting the conclusions of this article will be made available by the authors, without undue reservation.
